# The Escape from Malnutrition of Chilean Boys and Girls: Height-for-Age Z Scores in Late XIX and XX Centuries

**DOI:** 10.3390/ijerph181910436

**Published:** 2021-10-04

**Authors:** Javier Núñez, Graciela Pérez

**Affiliations:** 1 Economics Department, Faculty of Economics and Business, University of Chile, Santiago 832000, Chile; 2 Inter-American Development Bank, Washington, DC 20577, USA; grperez@fen.uchile.cl

**Keywords:** secular trends, stunting, height, anthropometry, Chile

## Abstract

We studied the trends of height-for-age (HAZ) Z scores by socioeconomic status (SES) groups of Chilean boys and girls aged 5–18 born between 1877 and 2001, by performing a meta-analysis of 53 studies reporting height-for-age sample data from which 1258 HAZ score datapoints were calculated using the 2000 reference growth charts for the US of the Centers for Disease Control and Prevention (CDC). We found stagnant mean and median HAZ scores of about −1.55 to −1.75 for the general population, and −2.2 to −2.55 for lower SES groups up to cohorts born in the 1940s. However, we found an upwards structural change in cohorts born after the 1940s, a period in which HAZ scores grew at a pace of about 0.25 to 0.30 HAZ per decade. Since this change happened in a context of moderate Gross Domestic Product (GDP) growth, high and persistent income inequality, and stagnant wages of the working class, we discuss the extent to which our findings are associated with the increase in public social spending and the implementation and expansion of a variety of social policies since the 1940s and early 1950s.

## 1. Introduction

In recent decades a large body of evidence has analyzed the secular growth in body size in many regions of the world in the last couple of centuries, which has expanded our understanding of the effects of improvements in environmental conditions on the standards of living and human welfare. Although a large share of the earlier literature focused on more developed or industrialized western countries, recently there has been many works devoted to the biological standard of living in the past in other regions, and in Iberian and Latin American countries in particular. This research agenda has produced a substantial long-term, comparative overview (mostly of the 19th and 20th centuries) of the biological well-being in the Iberian and Latin American region and its relationship with inequality and economic and social development [1,2,3,4,5]. Significant research has been done recently on various aspects of biological well-being especially in some of the largest countries of the region such as Argentina [6,7,8,9], Brazil [10,11], Chile [12,13,14,15,16], Colombia [17,18], Mexico [19,20,21,22], Peru [5] and Spain [23,24]. However, there is still less knowledge on three specific areas. First, there is less knowledge on the variations and determinants of secular growth in body size across socioeconomic groups within countries, especially in less developed countries, and how changes in living conditions and public policies may have heterogeneous impacts across socioeconomic segments and at different times [25,26]. Secondly, since the agenda has focused mostly on analyzing height in cm in adult populations, there is less knowledge on the patterns of secular growth of infants and adolescents in the past and their driving forces [26,27]. One important difference between studying heights of adults vs. children as a measure of biological well-being over time is that physical catch-up growth during teenage years and even early adulthood triggered by environmental improvements can partly compensate for early stunting and malnutrition, thus significantly reducing adult height deficits [28,29,30,31]. However, stunting and malnutrition in early years have a variety of irreversible long-term consequences in cognitive functions, brain development and other health problems even if children experience environmental improvements and physical catch-up growth at older ages [32,33,34,35,36]. This paper addresses these issues by employing an original dataset for analyzing the patterns of secular growth in height-for-age Z (HAZ) scores in boys and girls aged 5 to 18 years across socioeconomic groups in Chile, born between 1877 and 2001. We discuss this evidence in the context of the changes in economic growth, economic inequality and the development of social policies and public health in Chile during the XX century. This work builds on a previous article [26], which studies secular trends in the height of Chilean boys (in cm) and discusses the evidence in international comparative perspective. We expand this research specifically by: (i) adding height-for-age data of girls (who have been less studied in the literature, and who may have different trends in biological well-being than boys), (ii) adding a relevant amount of new studies that significantly expand the height-for-age of boys, and iii) transforming the resulting height-for-age sample data into HAZ scores using the CDC 2000 reference height growth charts for the US for boys and girls. This yields a consolidated dataset of 1258 datapoints of HAZ scores of samples of boys and girls aged 5 to 18 born between 1877 and 2001.

Using HAZ scores instead of height in cm has two advantages: first, it allows assessing the risk of stunting (chronic malnutrition) across boys and girls, as well as across ages. Accordingly, data can be combined and analyzed jointly with more statistical power. Secondly, HAZ scores can be more easily interpreted from a public health perspective, as HAZ scores are regularly used by governments, health experts and clinical practitioners to surveil growth and assess the pace of physical growth and the risk of stunting and malnutrition in individuals as well as in populations at large. Therefore, HAZ scores of children and adolescents reveal important information about the risk of stunting (chronic malnutrition) and the long-term human capital and biological well-being of individuals in the past.

A HAZ score is the deviation of the height for an individual or group of individuals from the mean/median height for the same age and sex in the reference population divided by the standard deviation for the reference population, in this case the 2000 CDC reference growth charts for the US (see [37] p. 7 for details). A HAZ score of −2 SD (approximately 3rd percentile of the reference growth charts) is accepted regardless of sex and age as a standard statistical cutoff point for stunting [33] and to determine the need for nutritional intervention [37].

The 2000 CDC reference charts are an improvement of the previous 1977 CDC charts designed to provide a reference for adequate growth of infants and teenagers of all major ethnic backgrounds in the US, including Hispanics and Mexican Americans (who share common Spanish and Amerindian ancestors with the Chilean population analyzed here). The recommendation of the 2000 CDC charts as a reference for growth regardless of race/ethnic considerations is based on evidence demonstrating that genetic effects on growth are small compared with the effects of environmental conditions, and that children of all major racial-ethnic groups provided with adequate nutrition, healthcare and living conditions appear to have similar growth patterns and potential [37].

The resulting dataset of HAZ scores is constructed from 53 studies published by health and education professionals between 1898 and 2014 that report individual height data or sample means of height-for-age of boys and girls aged 5–18 of different socioeconomic status (SES). This age group was chosen as is offers a more systematic coverage of height measurements of boys and girls from a variety of sources and socioeconomic groups, including pre-school facilities, public and private primary and secondary schools, orphanages and residential institutions, armed forces, and studies on the living conditions of poor and working-class children (often not attending schools in the first half of the XX century), among other sources. Even though the studies do not necessarily cover the same age groups within the 5–18 age range, HAZ scores are nevertheless comparable across ages and sex.

To estimate robust levels and trends in HAZ scores and address the effects of potential socioeconomic biases in the data, as in [26] we follow two complementary methodologies. First, we estimate levels and trends in HAZ scores from specific samples of boys and girls coming from 5 of the 53 studies, which report precise information about the socioeconomic status of the samples. These five studies either have an explicit method of socioeconomic characterization or provide a precise socioeconomic description of the individuals in the samples that can be employed to assign an upper or lower SES. These specific samples are then employed to calculate HAZ scores for upper and lower SES groups over time. Secondly, we run quantile regressions on quantiles 20 and 80 of the full dataset containing the 1258 HAZ scores in order to estimate secular growth in HAZ scores at the upper and lower quantiles of the distribution of HAZ scores, which are expected to be associated with upper and lower SES groups. We also run OLS and median regressions (50th quantile) as a robustness check to avoid the potential effect of outliers.

The next section describes the data and methods. The third section reports some stylized facts and the main results. Section 4 discusses the results in the context of improvements in environmental conditions in Chile during the XX century, and finally Section 5 concludes.

## 2. Data and Methods

Appendix A describes the 53 studies and their corresponding references, from which the 1258 datapoints of height-for-age sample means were obtained. In this dataset, 672 (53.4 percent) and 586 (46.6 percent) correspond to boys and girls, respectively. A subset of 447 samples of boys only coming from 38 studies was previously employed in [26]. Hence, this dataset significantly expands the dataset in [26] by including 15 new studies, adding original data for girls, and expanding the data for boys. The 1258 datapoints of height-for-age sample means in cm were transformed into HAZ scores using the LMS method and the CDC charts [37]. The LMS technique is based on three parameters estimated for a specific age and sex: the median (*M*), the generalized coefficient of variation (*S*), and the power in the Box–Cox transformation (*L*), which reflects the degree of skewness (generally low for height). A height of *x* cm is then expressed as $x=M{\left(1+LSZ\right)}^{\frac{1}{L}},\;L\neq 0$, where *Z* is the corresponding HAZ score (see [37], p. 7 for details). For example, a 14-year-old boy whose height is 158.9 cm has a corresponding HAZ score of −0.65 given the *L*, *M* and *S* values for 14-year-old boys provided in the CDC 2000 charts, that is, he is nearly 2/3 of a standard deviation below the median (or 50th percentile) of his 14-year-old peers (164.1 cm, see p. 171, [37]).

The studies presented in Appendix A show an important overall degree of diversity of age, sex, sources, and socioeconomic and regional strata of the population over time. The 5–18 age group was selected for exhibiting more density and diversity of institutional sources and socioeconomic groups over time. Most studies represent boys and girls from Santiago and other cities. However, smaller towns and rural areas are also represented in many studies. The density of studies is lower before the 1930s, and in this period the sources may underrepresent children from the poorest backgrounds, for example “street boys” [38] who often did not attend schools or other institutions from which many samples were obtained. Poor boys and girls are less likely to be underrepresented in the studies published after the 1930s because some of them perform explicit socioeconomic sampling procedures (such as [39] for example) and primary school enrollment was high and near universal in Chile since the late 1960s and early 1970s. Studies representing mixed socioeconomic groups were split into different samples whenever possible, in order to achieve wider socioeconomic diversity. In a few studies reporting individual height measurements, separate socioeconomic samples were determined from the available information.

Most studies in Appendix A have little details on the children’s ethnic/racial background, and therefore it was not possible to identify individual samples with specific ethnic groups (some of which seem historically penalized in height [15]). However, the samples are jointly expected to resemble Chile’s ethnic/racial composition dominated by a large majority of *Mestizo* population (mixed Spanish and Amerindian background) with small minorities of Amerindian and European descendants [40]. Immigration in Chile was low during the time span of this study and minimal in relation to the population [41]. Consequently, the estimated trends in HAZ scores would mostly be associated with changes in environmental conditions (health, nutrition, and sanitation among others) rather than changes in children’s genetic composition.

### 2.1. Levels and Growth of HAZ Scores from Specific Samples

Table 1 describes the samples of boys and girls by SES groups obtained from 5 of the 53 studies, selected for providing accurate socioeconomic information, and which are described in greater depth in [26] and the individual sources [39,42,43,44,45]. They comprise 126 of the total of 1258 sample HAZ scores of the full dataset (73 of boys and 53 of girls) and provide height-for-age sample means for upper and lower SES at three moments of the XX century: c. 1900, c. 1948 and 1991. These samples will be employed to estimate trends in HAZ scores for upper and lower SES groups over time.

### 2.2. Trends in HAZ Scores from Quantile Regressions on the Full Sample

Quantile regressions (see [46,47,48]) can provide estimates of the growth in HAZ scores across socioeconomic groups based on the ample evidence indicating that, as a consequence of inequalities in health and nutrition, in a given cohort height-for-age is associated with socioeconomic status. Accordingly, a sample’s HAZ score quantile in the conditional distribution is expected to be associated with the sample’s prevalent SES (and the corresponding underlying living conditions). This is consistent with the large HAZ score differences between socioeconomic groups presented in the next section. In this context, quantile regressions on HAZ scores of the full dataset would provide additional estimates of HAZ scores growth across socioeconomic groups exposed to different environmental (health and nutrition) conditions. Using the full sample, we estimate the following specification for the 20th, 50th and 80th quintiles, as well as by OLS;

(1) $$HAZ_{i}=\alpha +\beta Cohort_{i}+\gamma_{1}\;SEX_{i}+\gamma_{2}\;Location_{i}+\varepsilon_{i}$$

where $HAZ_{i}$ is the HAZ score of the mean height-for-age of individuals in sample *I* (which are all the same age), α is a constant term, $Cohort_{i}$ corresponds to the year of birth of individuals in sample *I*, $SEX_{i}$ is a dummy variable equal to 1 for boys, and $Location_{i}$ is a dummy variable equal to one for samples from Greater Santiago (Chile’s capital city). *β* measures the trend in HAZ scores and 10*β* yields the *decennial* secular growth in HAZ scores. Finally, $\varepsilon_{i}$ denotes the error term of the HAZ score of sample *I* associated with the sample’s prevalent SES (and health and nutrition status) and other non-observed variables affecting the samples’ HAZ score. We also estimate models with interactions between cohort and sex and location to control potential effects of changes in the composition of the dataset over time.

To identify structural changes in the trends of growth of HAZ scores over time, we also estimate OLS and quantile spline regressions, as follows:

(2) $$HAZ_{i}=\alpha +\beta Cohort_{i}+\delta D\left(Cohort_{i}-t^{*}\right)+\gamma_{1}Sex_{i}+\gamma_{2}Location_{i}+\varepsilon_{i}$$

where D=1 if $Cohort\geq t^{*}$ and 0 otherwise, where $t^{*}$ is the chosen knot for the spline regression (1930, 1940 and 1950). Therefore, in (2) β would now measure the secular trend in HAZ scores of cohorts born before year $t^{*}$, and δ would identify the change in the secular trend afterwards, such that growth after year $t^{*}$ is β+δ. Testing the hypothesis that secular growth in HAZ scores is constant for cohorts born before and after year $t^{*}$ (δ=0) allows testing a structural change in the growth of HAZ scores.

## 3. Results

### 3.1. Stylized Facts

**Figure 1:**
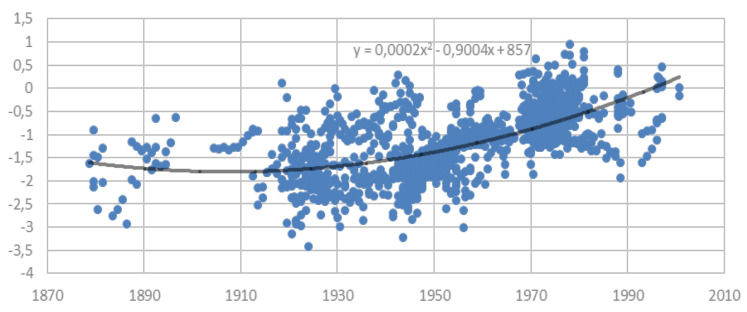
Height-for-Age Z scores (HAZ) of samples of Chilean boys and girls by birth cohorts (1877−2001), including a quadratic trend regression.

Figure 1 plots the 1258 HAZ scores of samples of Chilean boys and girls born between 1877 and 2001 according to their year of birth, including a fitted quadratic regression. Figure 1 suggests an overall increase in HAZ scores throughout the 120-year period, but at an accelerated pace during the second half of the XX century. The proportion of samples with average HAZ scores below −2 (conventional stunting threshold) is substantial up to approximately 1950, and zero after the early 1960s, a first indication of a significant improvement in the biological standard of living among low SES children since the mid-XX century. Note, however, that the datapoints correspond to mean HAZ scores of samples, and therefore the proportion of samples with HAZ scores below −2 cannot be considered as the prevalence of stunting over time, as the variation in height within samples is not observed in our data.

Table 2 presents summary statistics of the HAZ datapoints by decades of birth (the year the sample was taken minus age of children in the sample). It shows that the distribution of data within each decade is fairly unskewed as the mean and median HAZ scores are similar (except for the 1990s). The standard deviations of HAZ scores by decades are mostly in the range of 0.45 to 0.65 and always well below 1.0 as expected, which is approximately the standard deviation of HAZ scores when derived from individual data (if heterogeneity in the sample were similar to the reference data). Table 2 indicates that the mean and median HAZ scores are stable approximately in the range of −1.55 to −1.75 from the end of the XIX century up to 1950, suggesting a substantial prevalence of stunting and precarious biological standards of living in that period. Note, however, that the mean and median HAZ scores increase rapidly after approximately the late 1940s, up to about −0.4 HAZ towards the 1990s. Table 2 also indicates that the proportion of datapoints with HAZ scores less than −2 is stable in the range of 24 to 28 percent up to approximately 1950 and decline rapidly thereafter. There is only one sample out of 97 with a HAZ score less than −2 in the 1960s, and no samples under that threshold after 1970.

### 3.2. Socioeconomic Gaps and Trends in HAZ Scores from the Selected Samples

**Figure 2:**
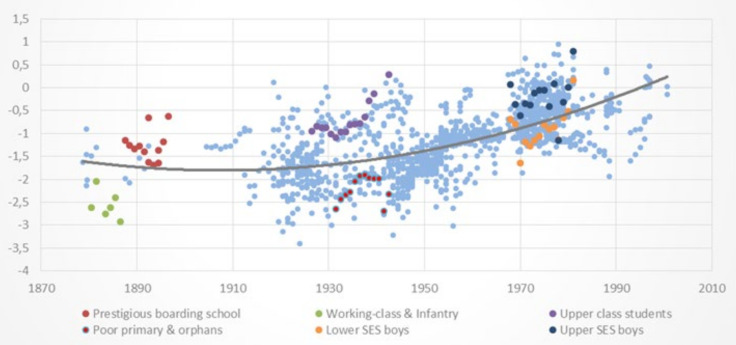
Height-for-Age Z scores (HAZ) of samples of Chilean boys and girls by birth cohorts (1877−2001). The six selected samples of boys of upper (three) and lower (three) socioeconomic status described in Table 1 are highlighted against the background of the full dataset.

**Figure 3:**
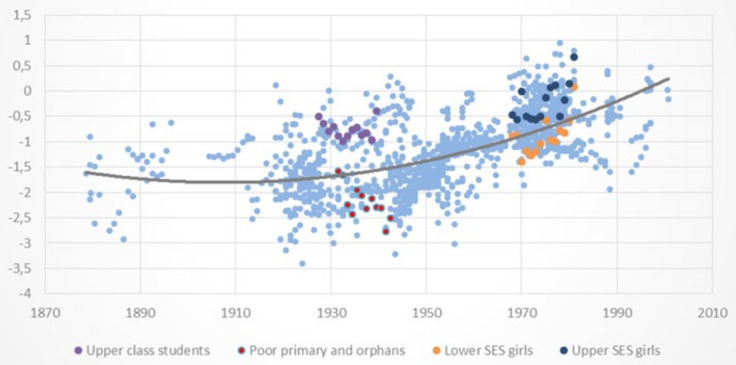
Height-for-Age Z scores (HAZ) of samples of Chilean boys and girls by birth cohorts (1877−2001). The four selected samples of girls of upper (two) and lower (two) socioeconomic status described in Table 1 are highlighted against the background of the full dataset.

Figure 2 and Figure 3 present the HAZ scores of the selected samples for boys and girls described in Table 1, respectively, plotted against the background of the 1258 datapoints of the full dataset. Both figures show that the samples selected to represent the upper and lower socioeconomic segments of boys and girls are respectively situated around the upper and lower sections or quantiles of the distribution of HAZ scores for similar birth cohorts, as expected. This suggests that the full dataset has a substantial overall representation of upper, middle, and lower SES groups over time. A large socioeconomic gap in HAZ scores is also visible throughout the 120-year span, providing support to the notion that HAZ scores quantiles in the conditional distribution of HAZ scores is associated with socioeconomic status, as argued earlier.

Table 3 reports mean HAZ scores of the 10 sex/SES selected group of samples described in Table 1 as well as the mean HAZ differences between upper and lower SES boys and girls c. 1900, c. 1948 and 1991, which correspond to cohorts born around 1880–90, 1928–1942 and 1968–1982, respectively.

Table 3 shows statistically significant differences in HAZ scores for upper and lower SES samples in the three cross-sectional periods. Socioeconomic differences in HAZ scores were large in c. 1900 (1.29 for boys) and in the late 1940s (1.50 and 1.42 for boys and girls, respectively), suggesting considerable differences in standards of living between upper vs. lower socioeconomic groups up to the 1940s. Table 3 also indicates some socioeconomic convergence in HAZ scores between c. 1948 and 1991, a period in which the gap decreased to 0.68–0.69 in 1991, suggesting a narrowing difference in living conditions between socioeconomic groups since the mid-XX century.

Table 4 reports the decennial growth in HAZ scores of upper and lower SES boys and girls aged 6-18, according to the HAZ scores in Table 3. The evidence indicates that children of both upper and lower SES experienced significant growth in HAZ scores during the XX century. However, the magnitude of the average decennial growth in HAZ scores for both socioeconomic groups is different, 0.13 and 0.18, for the upper and lower SES group, respectively.

Table 4 also confirms a faster pace of growth of HAZ scores in the second half of the XX century: upper-class boys and girls grew 0.12–0.13 HAZ per decade, respectively, and lower SES boys and girls show a substantial growth of 0.30–0.31 HAZ per decade, respectively. 

### 3.3. HAZ Scores Growth from OLS and 20, 50 and 80 Quantile Regressions

Table 5 reports OLS and quantile regressions of HAZ scores on birth cohorts, sex, and origin of the samples (Greater Santiago or otherwise), and interactions of both variables with year of birth. The OLS and median (Q50) regressions in Table 5 indicate estimates of decennial growth in HAZ scores of about 0.19–0.21 HAZ scores, respectively, which are stable across specifications. Samples from Greater Santiago have a significant HAZ score premium, which may reflect better health, nutrition, and sanitary conditions on average as well as potential socioeconomic biases in the samples. Although the interactive effects suggest a somewhat smaller rate of increase of HAZ scores over time for Greater Santiago, it does not appear to be stable in magnitude and statistical significance. Table 5 also indicates a small premium in HAZ scores for the female samples, but it is not robustly statistically significant, including the interactive term. BIC and AIC information criterion suggest as the preferred models the ones with the sex and Greater Santiago dummies, but without interactions with birth cohorts. Finally, quantile regressions report a decennial growth in HAZ scores of 0.21 for Q20 and 0.16 for Q80, close to the 0.18 and 0.13 reported in Table 4 for upper and lower SES boys, respectively, thus suggesting some degree of socioeconomic convergence in HAZ scores in the 120-year span.

Table 6 reports the annual growth in HAZ scores from spline OLS and quantile regressions as described by equation (2) in Section 2.2, which test structural changes in the growth of HAZ scores before and after birth cohorts 1930, 1940 and 1950. The corresponding regressions are included in Appendix B. Table 6 indicates that the growth in HAZ scores is statistically larger after all the three thresholds, according to the OLS and the median (Q50) regressions. In the case of the 1950 threshold, the OLS and the Q50 regressions provide similar decennial growths in HAZ scores of 0.09 before 1950 and 0.2–0.23 thereafter, which is consistent with the observed “kink” around 1950 discussed earlier as a stylized fact in Section 3.1. However, the AIC and BIC information criteria of the regressions in Appendix B are similar for the different breaks, suggesting that the structural change in the growth of HAZ scores may have begun in cohorts born before 1950. Note that the evidence is consistent with the acceleration in the growth of HAZ scores observed in the selected samples after 1948 in Table 4, which are cohorts born approximately in the 1930s and early 1940s.

## 4. Discussion

This article provides three main findings on the levels and growth of HAZ scores of Chilean boys and girls born since the 1880s. First, we find low levels of HAZ scores for the general population of boys and girls up to cohorts born approximately in the 1940s. Average and median HAZ scores were approximately in the range of −1.55 to −1.75 during this period, and HAZ scores of lower SES children were about −2.2 to −2.55. In addition, up to the mid-XX century the proportion of samples with mean HAZ scores lower than −2 were consistently in the range of 27 to 32 percent. Since in our data we only observe variations in HAZ scores between samples and not within samples, the proportion of stunted children with individual HAZ <-2 would have been presumably larger than that, suggesting widespread stunting in Chile up to cohorts born in the 1940s. 

Secondly, we find a large socioeconomic gap in HAZ scores in the range of 1.29 to 1.50 HAZ up to cohorts born in the 1930s and 1940s. This large gap is partly associated with the higher HAZ scores of upper SES boys and girls during the same period, in the range of −1.27 at the turn of the XX century and −0.76 towards the 1940s. Height-for-age of affluent Chilean boys was fairly high for a developing country, often similar although in some cases somewhat shorter than their North American and Western European counterparts in comparable periods [25,26,49,50,51]. Although there is not much literature to discuss socioeconomic gaps in HAZ scores in comparative historical perspective, our results are consistent with [26], which indicates a difference in height-for-age of boys in Chile in the first half of the XX century larger than socioeconomic height differences for contemporary teenage boys in Western countries [25,50,51,52,53]. 

These results suggest a high degree of inequality in living standards in Chile c. 1900, in line with the abundant reports of widespread poverty in Santiago and other cities associated with immigration from rural areas, which generated important sanitary, healthcare, and nutritional crises [54]. This led to a public discussion in Chile c. 1900 (known as *La Cuestión Social*) around the precarious living conditions of the poor [55,56], which were fueled by evidence of undernutrition and poor diet of the working class [57,58,59,60,61,62]. Low and stagnant HAZ scores up to the 1940s are also consistent with the socioeconomic statistics shown in Appendix B, namely very high infant mortality rates (270–200 per 1000 live births), low life expectancy (30–38 years), high income inequality (Gini coefficients 0.51–0.59), and negligible social policies and public social spending, especially in health, housing, and urban infrastructure. The low and stagnant HAZ scores up to the 1940s are also consistent with the modest economic growth during this period, with GDP per capita declining in comparison to the US, Western Europe, and Latin America. This is consistent with the evidence of sluggish real wages of the working class in Chile [63] and low economic growth in the first decades of the XX century (see Appendix C).

Third, we find robust evidence of a significant and generalized increase in HAZ scores since cohorts born in the 1940s, a pattern that is visible in a variety of methods, and across different socioeconomic groups. OLS and median regressions reveal a decennial growth in HAZ scores in the range of 0.24 to 0.29 and 0.22 to 0.31, respectively, depending on the birth cohort threshold. Similar increases in HAZ scores are observed in quantile regressions for quintiles 20 and 80, as well as in the analysis of selected samples in the same period, especially for the lower SES samples (0.3–0.31).

Our findings of a generalized increase in HAZ scores after the 1940s are also in line with the evidence of growth in height of Chilean adult men (soldiers), particularly in the second half of the XX century [16]. One revealing finding is that no samples with HAZ scores less than −2 are observed after the early 1960s, consistent with a rapid increase of HAZ among poor children. Of course, some stunting would have been prevalent due to unobserved individual variation in height-for-age within samples, but it does suggest a relevant drop in stunting after the 1950s. We also find some evidence of socioeconomic convergence in HAZ scores since approximately the 1930s, which is, however, stronger in the selected samples than in the quantile regressions (socioeconomic convergence in height is not a general rule, and both widening and converging class-differences in height have been reported [5,11,64,65,66,67,68,69,70]). 

Although there is not much evidence to compare the growth in HAZ scores of Chilean children and teenagers, our findings are in line with the substantial growth in height (in cm) in boys in industrialized countries [25,28,30,71,72,73].

Our finding of a rapid increase in HAZ scores in cohorts born approximately after the 1940s is consistent also with the considerable improvements in living standards shown in Appendix C in the same period, in particular the rapid reduction in infant mortality in Chile since the 1940s in comparison with the rest of Latin America, and the fast and steady increase in life expectancy from about 30 years at the beginning of the XX century to levels well above the average of Latin America, and close to some industrialized countries in the 1990s. Our results are also consistent with the fast pace of the demographic and epidemiological transitions that took place in Chile from about 1960 onwards [74,75], the nutritional transition after the 1930s [76], the considerable decline in the age of menarche in Chilean girls (due to earlier physical development) between 1940 and 1970 [77], and earlier maturation and growth spurt in Chilean boys in the second half of the XX century [26].

The economic and social statistics in Appendix C provide possible explanations for the structural break or “kink” observed in the rate of growth of HAZ scores in cohorts born around the 1940s and the rapid increase thereafter. First, the observed structural change is unlikely to be associated with changes in the rate of growth of GDP per capita, which shows a low decennial growth rate throughout most of the XX century, except for the exceptional 4 percent annual growth for the 1990s. To substantiate this claim, we tested structural changes in real GDP per capita in 1940 and in 1950 using the 1890–1990 annual data in [78], finding no evidence of statistically significant changes in the annual percent growth rate before vs. after either threshold (0.227, sd. 0.14 and 0.131, sd. 0.12, respectively). 

The mild improvements in income inequality observed between 1940 and 1970 may have played a role in improving living conditions and HAZ scores of poor Chilean children as suggested in the literature for other countries [25]. However, its impact is probably small or null given that income inequality remained high, in addition to the evidence indicating that real wages of the working class in Chile were in fact stagnant between 1940 and the mid-1960s [79].

As an alternative explanation, we conjecture that in a context of low GDP per capita growth, stagnant real wages of the working class, and high economic inequality, the improvements in children’s nutritional and health conditions are associated with the advent of an emergent welfare state since the mid-XX century, and its gradual expansion thereafter. Appendix C shows a considerable increase in public social spending as a fraction of GDP after the 1940s, and a relevant increase in the tax burden (tax revenues as a percentage of GDP) of four percentage points in the 1950s, and four to five extra percentage points in the 1960s and 1970s, which would have facilitated and provided the resources for the expansion of social policies even in a context of moderate economic growth. The increase in social public expenditure managed to finance landmark social policies in Chile [80,81,82]. Two relevant policies were the preventive medicine programs and the social protection programs for workers and their families established in 1938 (see [81,82]), and the pioneering foundation of the National Health Service (NHS) in 1952, a remarkable achievement compared to other countries of the Latin American region (see [83]). The NHS increasingly established a variety of programs that significantly improved the healthcare and nutrition of infants and their mothers even during pregnancy [62]. In addition, after the 1950s primary school enrollment increased rapidly, reaching near-universal coverage in 1970 (early in comparison with most countries of the region) after the 1965 education reform that declared primary education compulsory up to eighth grade. This expansion in primary education coverage and its extension in schooling years was combined with economic aid and feeding programs [84] that increased the nutrition of poor children and early teens. Investment in urban infrastructure including sewage and drinking water networks were implemented at a faster pace after the 1950s and 1960s in urban areas [85], in a context of substantial population growth in larger cities associated with increasing industrialization [86]. As a result, in the 1980s urban sewage was widespread and the coverage of households with safe drinking water was near universal (see Appendix C). Another pioneering initiative was the Corporation for Infant Nutrition (CONIN), a public–private partnership founded in 1974 with the aim of implementing a 20-year national plan to eradicate undernutrition [87], a model that later was implemented in other counties of the region due to its success. All these policies combined promoted better nutrition, healthcare and sanitation, less exposure to infections and disease, and better living conditions for poorer Chilean infants and adolescents, especially in urban areas, despite low economic growth and high economic inequality.

## 5. Conclusions

This paper develops a novel methodology based on a meta-analysis of 53 studies reporting height-for-age of Chilean boys and girls to study the levels and trends in HAZ scores in the late XIX and XX centuries. As HAZ scores can be interpreted from a public health perspective as an indicator of risk of stunting (chronic malnutrition), this methodology allows studying malnutrition and stunting in ages that are critical for the biological, cognitive, and social development of children and their long-term human capital and welfare.

We found low and stagnant HAZ scores in cohorts born since the end of the XIX century and up to the 1940s (in the range of −1.55 to −1.75 on average, and −2.2 to −2.55 for poor children), suggesting a context of widespread stunting, especially among the poor. We suggest that these findings are associated with the ample evidence indicating very precarious environmental conditions in Chile during this period, reflected in low standards of living, especially in health, nutrition, and sanitation, paired with high economic inequality, and negligible social public expenditure.

However, we also find a robust and generalized upwards structural change in HAZ scores since the cohorts born approximately in the 1940s, in which HAZ scores grew considerably (about 0.25 to 0.3 HAZ per decade). Since this upwards structural change occurred in a context of low to moderate growth in GDP per capita, high and persistent income inequality, and stagnant real wages of the working class, we suggest that our findings are associated with the steady increase of public social expenditure and the implementation and steady expansion of a variety of social policies in health, nutrition, housing, sanitation, public infrastructure and education in Chile since the 1940s.

We suggest that a similar methodology to the one employed here based on a meta-analysis of sources reporting height-for-age in the past can be performed for other countries. This would allow studying in comparative perspective the extent to which differences in children’s HAZ scores between countries and changes in HAZ scores of children in the past are associated with environmental improvements led by economic development, changes in economic inequality, and/or development of social policies, among other factors.

## Figures and Tables

**Table 1:** Selected studies providing accurate information of children‘s socioeconomic background.

Study	Sex	SES	Year	Description and Location	Ages
[42]	Only Boys	Lower	1898	Working-class boys, infantry, poor primary school students from low SES neighborhoods in Santiago and Valparaíso.	11–18
[43]	Only Boys	Upper	1907	Upper and upper-middle class students from a privileged boarding school in Santiago (INBA).	10–18
[44]	Boys and Girls	Lower	1946	Poor (*clase baja*), working-class children from Santiago’s lower SES *Quinta Normal* Municipality and from *Ciudad del Niño* orphanage in Santiago.	6–19
[45]	Boys and Girls	Upper	1948	Affluent *Humanidades* students (in preparation for university/tertiary education), and students from privileged private and public schools in *Servicio Médico Escolar* in Santiago.	6–16
[39]	Boys and Girls	Upper and Lower	1990	Representative samples of students by SES, Greater Santiago. Socioeconomic samples derived from a Graffar scale based on the schooling and occupation of the household head and dwelling characteristics.	6–18

**Table 2:** Descriptive statistics of samples, by birth decades.

Height-for-Age (Z Scores), Full Sample					HAZ Score < −2	
Birth Decade	Mean	Median	s.d.	Total Samples	Samples HAZ < −2	%
1877–1910	−1.626	−1.443	0.563	34	9	26.5
1911–1920	−1.579	−1.765	0.671	30	8	26.7
1921–1930	−1.723	−1.736	0.606	168	55	32.7
1931–1940	−1.559	−1.773	0.693	99	29	29.3
1941–1950	−1.630	−1.738	0.635	207	56	27.1
1951–1960	−1.387	−1.345	0.487	210	19	9.1
1961–1970	−0.890	−0.930	0.423	97	1	1.0
1971–1980	−0.564	−0.557	0.459	326	0	0.0
1981–1990	−0.680	−0.604	0.600	66	0	0.0
1991–2001	−0.428	0.001	0.646	21	0	0.0
Total				1258	177	

**Table 3:** Mean HAZ scores of selected upper and lower SES samples, boys and girls.

	Upper SES			Lower SES			Difference Upper vs. Lower SES		
	1907	1948	1991	1898	1948	1991	c. 1900	1948	1991
	(1)	(2)	(3)	(4)	(5)	(6)	(1–4)	(2–5)	(3–6)
Boys HAZ	−1.27	−0.71	−0.20	−2.55	−2.21	−0.89	1.29 ***	1.50 ***	0.69 ***
S.D.	(0.34)	(0.38)	(0.44)	(0.31)	(0.28)	(0.42)			
Girls HAZ	n.a.	−0.76	−0.21	n.a.	−2.19	−0.89	n.a.	1.42 ***	0.68 ***
S.D.	-	(0.17)	(0.37)	-	(0.34)	(0.37)			
Boys observations	12	15	14	6	12	14			
Girls observations	-	13	14	-	12	14			
Total individuals contained in samples	821	3706	532	73	401	871			

Standard deviation in parenthesis. *** *p* < 0.01.

**Table 4:** Decennial HAZ growth, boys and girls aged 6–18, upper vs. lower SES.

	Upper SES			Lower SES		
	1907–1948	1948–1991	1907–1991	1899–1948	1948–1991	1899–1991
Boys	0.14 ***	0.12 ***	0.13 ***	0.07 **	0.31 ***	0.18 ***
Girls	n.a.	0.13 ***	n.a.	n.a.	0.30 ***	n.a.

*** *p* < 0.01, ** *p* < 0.05.

**Table 5:** OLS and quantile regressions on HAZ scores, Chilean boys and girls, cohorts 1887–2001.

Height for Age Z-Scores	OLS	OLS	OLS	Q50	Q50	Q50	Q20	Q80
Year of Birth	0.019 ***	0.019 ***	0.022 ***	0.021 ***	0.021 ***	0.026 ***	0.021 ***	0.016 ***
	(0.001)	(0.001)	(0.003)	(0.001)	(0.001)	(0.002)	(0.001)	(0.001)
Greater Santiago = 1		0.538 ***	0.721 ***		0.512 ***	0.888 ***	0.380 ***	0.745 ***
		(0.033)	(0.240)		(0.042)	(0.185)	(0.042)	(0.063)
Male = 1		−0.044	0.165		−0.016	0.148	−0.131 ***	−0.026
		(0.031)	(0.135)		(0.036)	(0.131)	(0.036)	(0.054)
Year of Birth * Santiago			−0.002			−0.005 **		
			(0.003)			(0.002)		
Year of Birth * Male			−0.003 *			−0.002		
			(0.002)			(0.002)		
Constant	−2.626 ***	−2.983 ***	−3.248 ***	−2.832 ***	−3.173 ***	−3.582 ***	−3.511 ***	−2.466 ***
	(0.070)	(0.069)	(0.220)	(0.078)	(0.070)	(0.185)	(0.071)	(0.107)
Observations	1258	1258	1258	1258	1258	1258	1258	1258
R-squared	0.335	0.435	0.437					
Pseudo R-squared				0.202	0.281	0.282	0.283	0.238
AIC	2265	2064	2063					
BIC	2276	2085	2094					

Robust standard errors in parentheses. *** *p* < 0.01, ** *p* < 0.05, * *p* < 0.1.

**Table 6:** Annual HAZ scores growth from OLS and quantile spline regressions.

Dependent Variable: HAZ Score	OLS	Q20	Q50	Q80
Annual growth birth cohorts 1877–2001	0.019 ***	0.021 ***	0.020 ***	0.016 ***
	(0.001)	(0.001)	(0.001)	(0.001)
Annual growth before 1950	0.009 ***	0.014 ***	0.009 ***	0.004 *
	(0.002)	(0.002)	(0.002)	(0.002)
Additional annual growth after 1950	0.020 ***	0.011 ***	0.023 ***	0.026 ***
	(0.003)	(0.003)	(0.003)	(0.004)
Annual growth before 1940	0.004 *	0.009 ***	−0.001	0.004
	(0.002)	(0.002)	(0.002)	(0.003)
Additional annual growth after 1940	0.022 ***	0.015 ***	0.032 ***	0.020 ***
	(0.003)	(0.003)	(0.003)	(0.005)
Annual growth before 1930	−0.001	−0.004	−0.007 ***	0.003
	(0.003)	(0.003)	(0.003)	(0.005)
Additional annual growth after 1930	0.025 ***	0.028 ***	0.034 ***	0.018 ***
	(0.003)	(0.004)	(0.003)	(0.005)
Observations	1258	1258	1258	1258

Note: Each point estimate is from a separate regression presented in Appendix B where HAZ score is the dependent variable. Coefficients after the threshold represent the change in the slope of year of birth from the preceding interval. All regressions condition on location of samples (Greater Santiago or otherwise) and sex. Robust standard errors in parentheses. *** *p* < 0.01, * *p* < 0.1.

## Appendix A

**Table A1:** Studies providing height-for-age of samples of boys and girls, Chile 1893–2014.

No	Study/Report	Year of Survey	Age	Sex (No. Observations)	Samples’ Locations	Description and SES
1	[88] Chilean Army (1893–1897)	1893–97	14–17	M(41)	Central/Southern Chile (urban and rural)	Draft soldiers, Mixed SES
2	[42] Moraga del Hoyo (1899)	1898	10–20	M(159)	Santiago and Valparaíso (urban)	Mixed SES
3	[43] Matus Zañartu (1911)	1907	10–20	M(881)	Santiago (urban)	Students, IMBA boarding school
4	[89] Matus Ugarte (1930)	1924	6–20	M(9750)	Santiago (urban)	Upper-middle class students
5	[90] Del Solar (1929)	1928	6–15	M(9244), F(11668)	Santiago and Valparaíso (urban)	Students, mixed SES
6	[58] Jimenez (1934) in Allende (1939)	1934	12	M(400)	Santiago (urban)	Primary school students
7	[91] De María (1934) in Riquelme (1942)	1934	6–15	M, F	Greater Santiago (urban)	Primary school Students
8	[92] Mardones and Sépulveda (1936)	1936	6–16	F(554)	Santiago (urban)	Schoolchildren, Mixed SES
9	[93] Ortega (1935)	1935	5–14	M(272), F(603)	Santiago (urban)	Students, mixed SES
10	[91] Riquelme (1942)	1940	6–15	M, F, (202)	Conchalí (rural)	Students sons of peasants/workers
11	[91] Bustamante (1937) in Riquelme (1942)	1937	7–15	M, F	Santiago (urban)	Primary school students
12	[94] Parry (1939)	1937	12–18	M(3677)	Santiago (urban)	Well-off students
13	[95] Alvial (1940)	1940	9–17	F(1006)	Greater Concepción (urban, some rural)	Primary school students
14	[96] Illanes and Correa (1944)	1944	7–14	M(56), F(52)	Santiago (urban)	Schoolchildren
15	[97] Giacaman (1945)	1945	8.9, 17	M(68), F(69)	Greater Concepción (urban, some rural)	Mixed SES
16	[98] Durán (1946)	1946	7–16	M(391), F(309)	Santiago (urban)	Orphans in residential institutions
17	[44] Mesa (1948)	1947	5–20	M(3720),F(1099)	Santiago (urban)	Well-off students
18	[45] Royo (1948)	1948	3–16	M(608), F(478)	Santiago (urban)	Students, sons of workers
19	[99] Burkhardt (1949) in Cusminsky (1968)	1949	10–15	M, F, (6653)	Santiago (urban)	Primary school students
20	[99] Muñoz (1950) in Cusminsky (1968)	1950	7–13	M(500)	Santiago (urban)	Primary school students
21	[100] Jadresic (1950)	1950	16–20	M(90)	Santiago (urban)	Students, IMBA boarding school
22	[101] Hernández (1953)	1952	19	M(432)	Central Chile (urban and rural)	Soldiers
23	[99] Saavedra (1954) in Cusminsky (1968)	1954	6–12	M, F,535	San Miguel (urban some rural)	Working-class children
24	[99] Viel (1954), in Cusminsky (1968)	1954	9–14	M(5989)	Santiago (urban)	Well-off students
25	[102] Norris Cumming (1958)	1958	5–14	M(18809), F(12336)	Santiago (urban)	Students, sons of employees
26	[103] Stegen and Barros (1960)	1957	0–15	M(935), F(873)	Valparaíso (urban and rural)	Poor, working-class children
27	[60] ICNND, US (1961)	1961	5–16	M(22494), F(20386)	Northern, Central and Southern Chile (urban, some rural)	Students, mixed SES
28	[104] Tapia et al. (1963)	1962	7–14	M(1143), F(1142)	Southern Santiago (urban)	Students, lower SES
29	[105] Montoya and Ipinza (1964)	1963	5–17	M(210), F(194)	Santiago (urban)	Well-off pre-school students
30	[106] Monckeberg et al. (1967)	1966	6	M(16)	Curicó Province (urban and rural)	Pre-school children, mixed SES
31	[107] Kram and Soto (1967)	1967	6–13	M(61), F(53)	Bahía Mansa, Osorno (rural)	Primary and secondary students
32	[108] Concha et al. (1969)	1968	12–16	M(77), F(78)	Santiago (urban)	School students, lower SES
33	[109] Rona and Pierret (1973)	1970	10–17	F(400)	Santiago (urban)	Public and private school students
34	[77] Rona (1975)	1970	10–16	F(354)	Santiago (urban)	Public and private school students
35	[110] Araya et al. (1974)	1974	6–20	M(567), F(500)	Greater Santiago (urban)	Mixed SES students
36	[111] Avendaño et al. (1974)	1971–72	6–20	M(1347), F(1238)	North Santiago (urban)	Middle SES students
37	[112] Valenzuela and Avendaño (1979)	1971–72	6–20	M(2257), F(1878)	North Santiago (urban)	Middle SES students
38	[113] Patri et al. (1984)	1976	5–6	M,F (438)	North Santiago (urban)	Middle SES pre-schoolers
39	[114] Beas et al. (1986)	1986	8–18	M(263)	Greater Santiago (urban)	Upper and middle SES students
40	[115] Mauricci et al. (1986)	1985	11–16	M(270), F(394)	Greater Santiago (urban)	Secondary school students
41	[116] Moreno (1994)	1988–94	10–17	M(583), F(519)	Antofagasta (urban and rural)	Mixed SES students
42	[117] Valenzuela and Avendaño (1988)	1971–72	5–20	M(500), F(500)	North Santiago (urban and rural)	Middle SES students
43	[118] Youlton and Valenzuela (1990)	1985	0–17	M(7500), F(7500)	Greater Santiago (urban)	Upper and upper-middle SES
44	[39] Ivanovic et al. (1991)	1986–87	5–18	M(2241), F(2110)	Greater Santiago (Metropolitan Region, urban, some rural)	Mixed SES students
45	[119] Burrows et al. (1992)	1985	6–15	M(2741), F(2816)	Greater Santiago (urban)	Mixed SES students
46	[120] Sanz (1993)	1993	7–15	M(413), F(366)	Valdivia (urban and rural)	Students
47	[121] Amigo et al. (1995)	1995	7	M(2292), F(2320)	Greater Santiago (urban)	Upper-middle SES Primary students
48	[122] Valenzuela (1997)	1996	7–20	M(500), F(500)	Northern Santiago (urban)	Lower-middle SES students
49	[123] Moreno (1998)	1990	11–14	M(456), F(447)	Antofagasta Region (urban and rural)	Mixed SES students
50	[124] Burrows et al. (2004)	2003	6–15	M(1561), F(1227)	Greater Santiago (urban)	Mixed SES students
51	[125] Alarcón and Atalah (2009)	2007	6.3, 6.4	M(225), F(201)	Vicuña, Northern Chile (urban)	Primary students, mixed SES
52	[126] Atalah et al. (2012)	1997 2005	6, 15	M(56737), F(61008)	All 15 regions of Chile (urban and rural)	School students
53	[127] Vargas et al. (2014)	2014	14–18	M(13580), F(6390)	All 15 regions of Chile (urban and rural)	Students, mixed SES

## Appendix B

**Table A2:** OLS and quantiles regressions with breaks in 1930.

Height-for-Age Z Scores	OLS	Q20	Q50	Q80
Birth Before 1930	−0.001	−0.004	−0.007 ***	0.003
	(0.003)	(0.003)	(0.003)	(0.005)
Birth After 1930	0.025 ***	0.028 ***	0.034 ***	0.018 ***
	(0.003)	(0.004)	(0.003)	(0.005)
Greater Santiago = 1	0.488 ***	0.342 ***	0.441 ***	0.675 ***
	(0.033)	(0.043)	(0.039)	(0.067)
Male = 1	−0.065 **	−0.123 ***	−0.028	−0.080
	(0.030)	(0.037)	(0.033)	(0.057)
Constant	−2.063 ***	−2.256 ***	−1.853 ***	−1.909 ***
	(0.140)	(0.148)	(0.133)	(0.229)
Observations	1258	1258	1258	1258
R-squared	0.467			
Pseudo R-squared		0.300	0.309	0.257

Robust standard errors in parentheses. *** *p* < 0.01, ** *p* < 0.05.

**Table A3:** OLS and quantiles regressions with breaks in 1940.

Height-for-Age Z Scores	OLS	Q20	Q50	Q80
Birth Before 1940	0.004 *	0.009 ***	−0.001	0.004
	(0.002)	(0.002)	(0.002)	(0.003)
Birth After 1940	0.022 ***	0.015 ***	0.032 ***	0.020 ***
	(0.003)	(0.003)	(0.003)	(0.005)
Greater Santiago = 1	0.458 ***	0.341 ***	0.375 ***	0.628 ***
	(0.035)	(0.042)	(0.038)	(0.067)
Male = 1	−0.053 *	−0.102 ***	−0.026	−0.058
	(0.030)	(0.035)	(0.032)	(0.056)
Constant	−2.222 ***	−2.830 ***	−1.994 ***	−1.886 ***
	(0.124)	(0.125)	(0.112)	(0.197)
Observations	1258	1258	1258	1258
R-squared	0.466			
Pseudo R-squared		0.296	0.312	0.261

Robust standard errors in parentheses.*** *p* < 0.01, * *p* < 0.1.

**Table A4:** OLS and quantiles regressions with breaks in 1950.

Height-for-Age Z Scores	OLS	Q20	Q50	Q80
Birth Before 1950	0.009 ***	0.014 ***	0.009 ***	0.004 *
	(0.002)	(0.002)	(0.002)	(0.002)
Birth After 1950	0.020 ***	0.011 ***	0.023 ***	0.026 ***
	(0.003)	(0.003)	(0.003)	(0.004)
Greater Santiago = 1	0.428 ***	0.345 ***	0.370 ***	0.545 ***
	(0.038)	(0.042)	(0.041)	(0.063)
Male = 1	−0.047	−0.114 ***	-0.017	−0.026
	(0.030)	(0.034)	(0.033)	(0.051)
Constant	−2.379 ***	−3.077 ***	−2.421 ***	−1.797 ***
	(0.109)	(0.107)	(0.105)	(0.160)
Observations	1258	1258	1258	1258
R-squared	0.463			
Pseudo R-squared		0.288	0.304	0.274

Robust standard errors in parentheses. *** *p* < 0.01, * *p* < 0.1.

## Appendix C

**Table A5:** Selected social and economic indicators, Chile, c. 1900 to 2000.

	1905–1907	1920	1930	1940	1950	1960	1970	1980	1990	2000
Social Public Spending/GDP (%) ^(1)^	1.1	1.0	2.7	3.6	5.2	8.6	10.5	10.3	12.9	16.6
Education	1.1	1.0	1.7	1.9	2.4	2.6	3.3	3.5	2.5	4.0
Health	0.0	0.0	1.0	0.6	0.7	2.2	2.0	3.2	2.0	2.8
Pensions and Social Welfare	0.0	0.0	0.0	0.7	1.4	3.1	2.6	3.0	6.3	6.9
Housing and Urban Planning	0.0	0.0	0.0	0.4	0.6	0.6	2.1	0.6	1.0	1.3
GDP per capita (pc), 2011 $ ^(2)^	4485	4248	4876	5177	5880	6781	8195	9024	10,203	15,212
Annual GDP pc growth in decade		−0.5	1.4	0.6	1.3	1.4	1.9	1.0	1.2	4.1
GDP pc relative to US ^(2)^	0.47	0.42	0.46	0.43	0.39	0.38	0.34	0.30	0.28	0.33
GDP pc relative to W. Europe ^(2)^	0.87	0.87	0.76	0.72	0.81	0.62	0.51	0.43	0.40	0.47
GDP pc relative to L. America ^(2)^	2.04	1.82	1.81	1.71	1.58	1.43	1.30	1.03	1.25	1.49
Tax Burden (Tax Revenues/GDP) ^(3)^	7.4	5.8	10.0	9.8	9.6	13.6	17.3	18.7	17.0	16.0
Life Expectancy at Birth (Years) ^(4)^	30.0	31.0	35.0	38.0	54.8	58.5	63.3	70.7	74.3	77.7
Infant Mortality Rate (IMR) ^(5)^	273.8	244.4	219.6	200.7	140.6	119.3	73.7	31.1	15.9	9.3
(per 1,000 live births)										
IMR Latin America ^(6)^					126	101	80	59	43	28
Urban Drinking Water (%) ^(7)^						44.8	66.5	91.4	97.4	99.2
Urban Sewage (%) ^(8)^						21.3	31.1	67.4	82.6	92.1
Rural Drinking Water (%) ^(8)^						9.5	34.2	44.2	79.9	75.3
GINI Index ^(9)^	50.9	56.3	58.6	57.2	0.52	0.54	0.49	0.53	0.55	0.56

Social spending figures for 1940 and 1950 are interpolations from 1935–1945, and 1945–1955 of the source, respectively. ^(1)^ 1905–1907 to 1980; [80]; [128]: p. 36. 2000 figure is 1999 figure in source. ^(2)^ [78]: data for 1905-7 correspond to 1910 data in source. ^(3)^ [129]. ^(4)^ [129]. ^(5)^ With respect to *corrected births* in [129]. ^(6)^ [130]: approximations and interpolations from 5-year data in the source. ^(7)^ [131]: 1960 figure corresponds to 1963 in source. ^(8)^ [131]: 2000 figure is 1997 figure in source. ^(9)^ [129]: Code GINIROD (Rodríguez).
